# Supplementary material for: Impact of Image Context on Deep Learning for Classification of Teeth on Radiographs
Source: J Clin Med. 2021 Apr 12;10(8):1635. doi: 10.3390/jcm10081635 (PMC8068972; doi:10.3390/jcm10081635)
Supplement: Supplementary file 1 [file jcm-10-01635-s001.pdf]

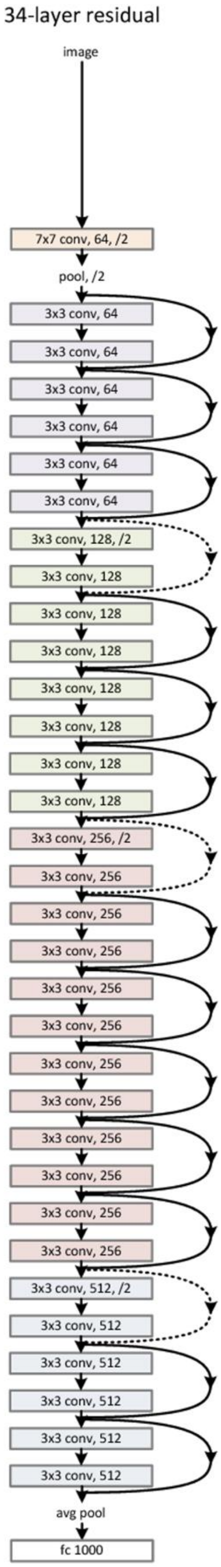

Figure 1. Architecture of ResNet-34 network with 34 parameter layers. Curved arrows symbolize identity mapping. Dotted shortcuts represent an increase of the dimensions.
